# Supplementary material for: Transcriptome size matters for single-cell RNA-seq normalization and bulk deconvolution
Source: Nat Commun. 2025 Feb 1;16:1246. doi: 10.1038/s41467-025-56623-1 (PMC11787294; doi:10.1038/s41467-025-56623-1)
Supplement: Supplementary file 1 — Supplementary Information [file 41467_2025_56623_MOESM1_ESM.pdf]

Supplementary Information to

Transcriptome size matters for single-cell RNA-seq normalization and bulk deconvolution

Lu, et al.

Supplementary Figures 1-16

Supplementary Tables 1-3

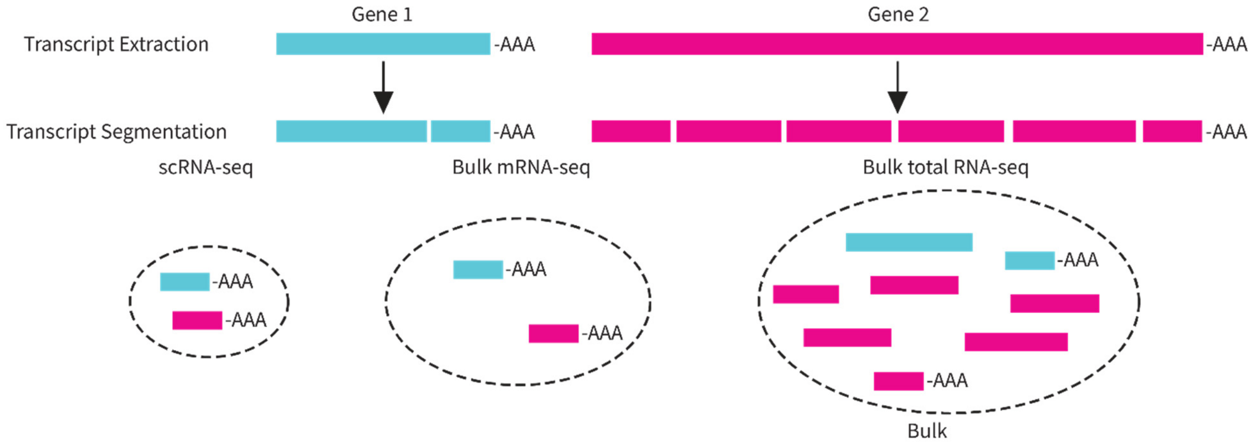

	Transcript count	Segment count	scRNA-seq raw count	mRNA-seq raw ocunt	mRNA-seq CPM	totalRNA-seq raw count	totalRNA-seq TPM
Gene 1	**1**	**2**	**1**	**1**	c_1	**2**	c_2
Gene 2	**1**	**6**	**1**	**1**	c_1	**6**	c_2

Supplementary Fig. 1 | Illustrates of different RNA-seq techniques. Illustrates the readouts for scRNA-seq, bulk mRNA-seq, and bulk total RNA-seq data that were subjected to different normalization methods.

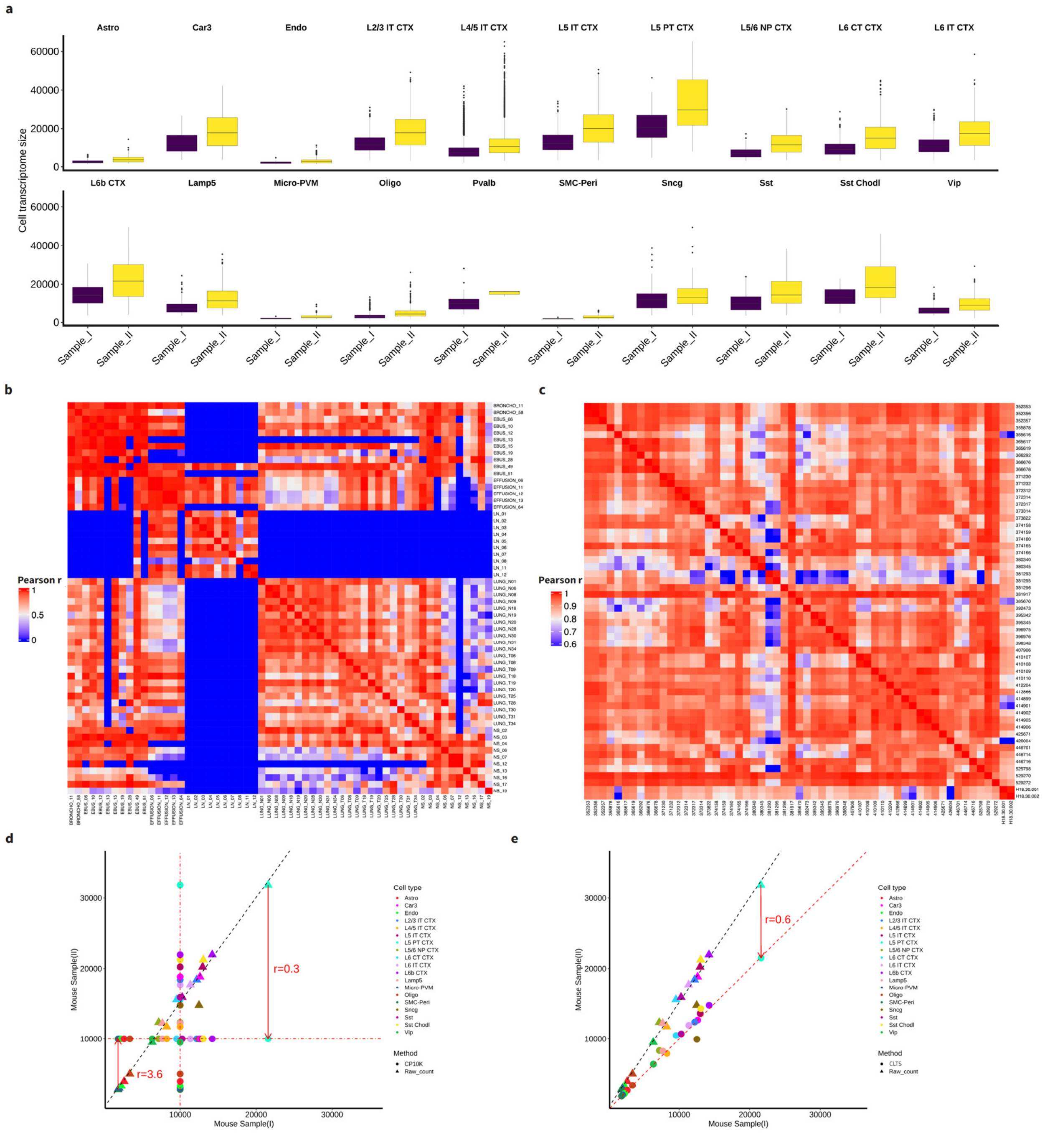

Supplementary Fig. 2 | Cell type transcriptome sizes and their linear correlations. a, Transcriptome sizes of different types of cells in mouse brain Sample_I and Sample_II. **b, c,** Heatmap showcasing the Pearson correlation coefficients of mean cell type transcriptome size for any two samples that share a minimum of four cell types, with each cell type having at least 10 cells in both samples in scRNA-seq

dataset of GSE131907 (**b**) and scRNA-seq dataset of mouse samples from the Allen Brain Map (**c**). **d, e,** Illustration of the changes of transcriptome size means of all cell types under CP10K (**d**) and CLTS normalizations (**e**), respectively. For CP10K normalization (**d**), transcriptome size means of different cell types were amplified or suppressed with uneven ratios. For CLTS normalization (**e**), transcriptome size means of different cell types were amplified or suppressed with almost equal ratio. In the box plots presented in this figure, the values are depicted as the median, represented by the middle line, and the 25th and 75th percentiles, represented by the box. In Sample_I of (**a**): L4/5 IT CTX (n = 10263), L6 CT CTX (n = 3586), L2/3 IT CTX (n = 2552), L6 IT CTX (n = 1671), L5 IT CTX (n = 1665), L5/6 NP CTX (n = 641), L6b CTX (n = 447), Oligo (n = 408), Vip (n = 380), Lamp5 (n = 362), Sst (n = 331), Astro (n = 206), Sncg (n = 98), Car3 (n = 91), L5 PT CTX (n = 85), Pvalb (n = 53), Sst Chodl (n = 50), Endo (n = 45), Micro-PVM (n = 26), SMC-Peri (n = 12). In Sample_II of (**a**):L4/5 IT CTX (n = 19932), L6 CT CTX (n = 4605), L2/3 IT CTX (n = 3983), L6 IT CTX (n = 1963), L5 IT CTX (n = 1857), L5/6 NP CTX (n = 1107), L6b CTX (n = 481), Oligo (n = 708), Vip (n = 414), Lamp5 (n = 456), Sst (n = 161), Astro (n = 461), Sncg (n = 59), Car3 (n = 142), L5 PT CTX (n = 37), Pvalb (n = 3), Sst Chodl (n = 33), Endo (n = 138), Micro-PVM (n = 125), SMC-Peri (n = 75). Source data are provided as a Source Data file.

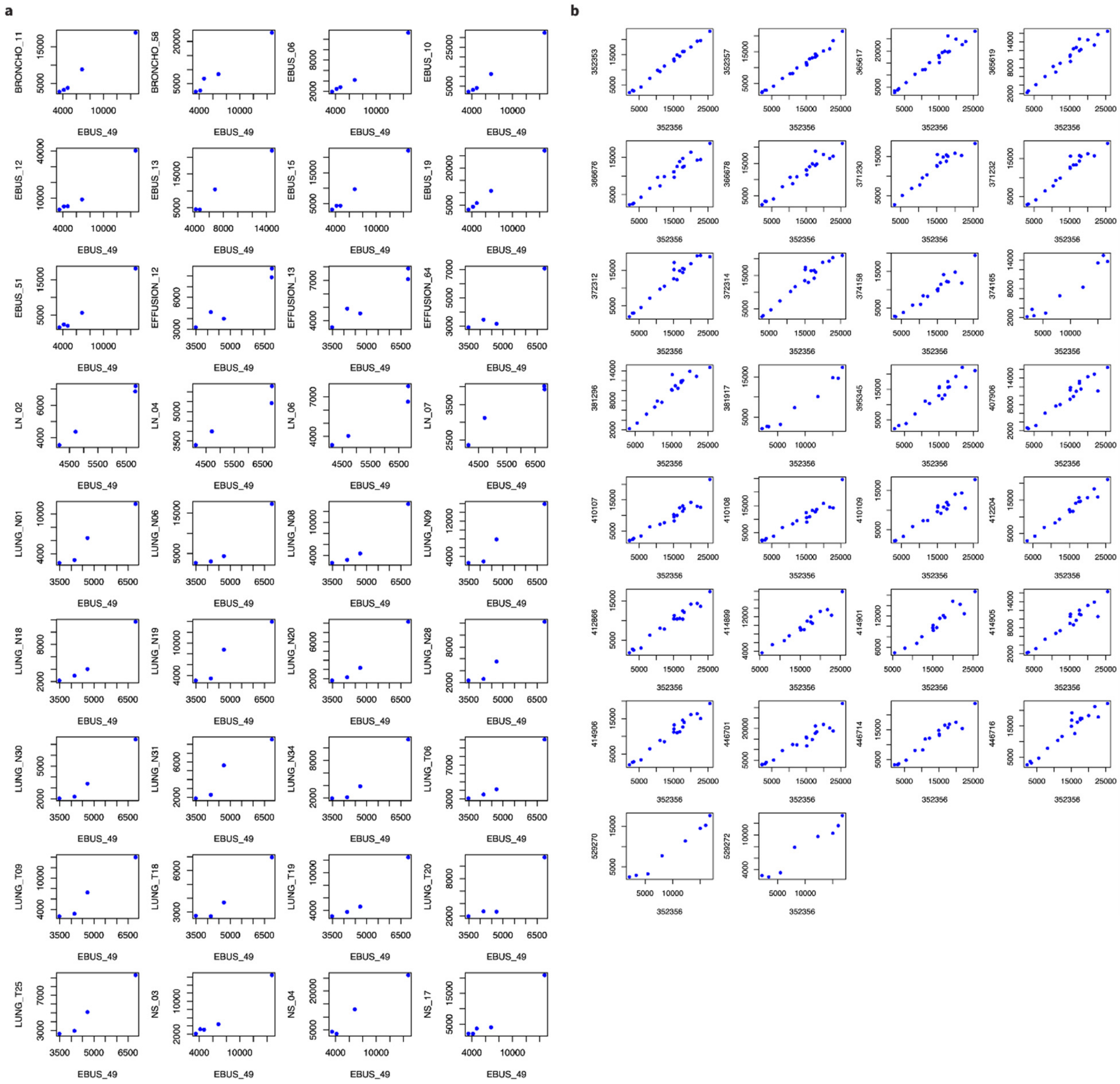

Supplementary Fig. 3 | Linear correlation between transcriptome size averages across various cell types in different samples. a, b, Scatter plots of mean cell type transcriptome sizes between different samples. **a,** Between lung cancer sample EBUS_49 and other lung cancer samples in GSE131907. **b,** Between mouse sample 352356 and other mouse samples in the scRNA-seq dataset from the Allen Brain Map. Source data are provided as a Source Data file.

a

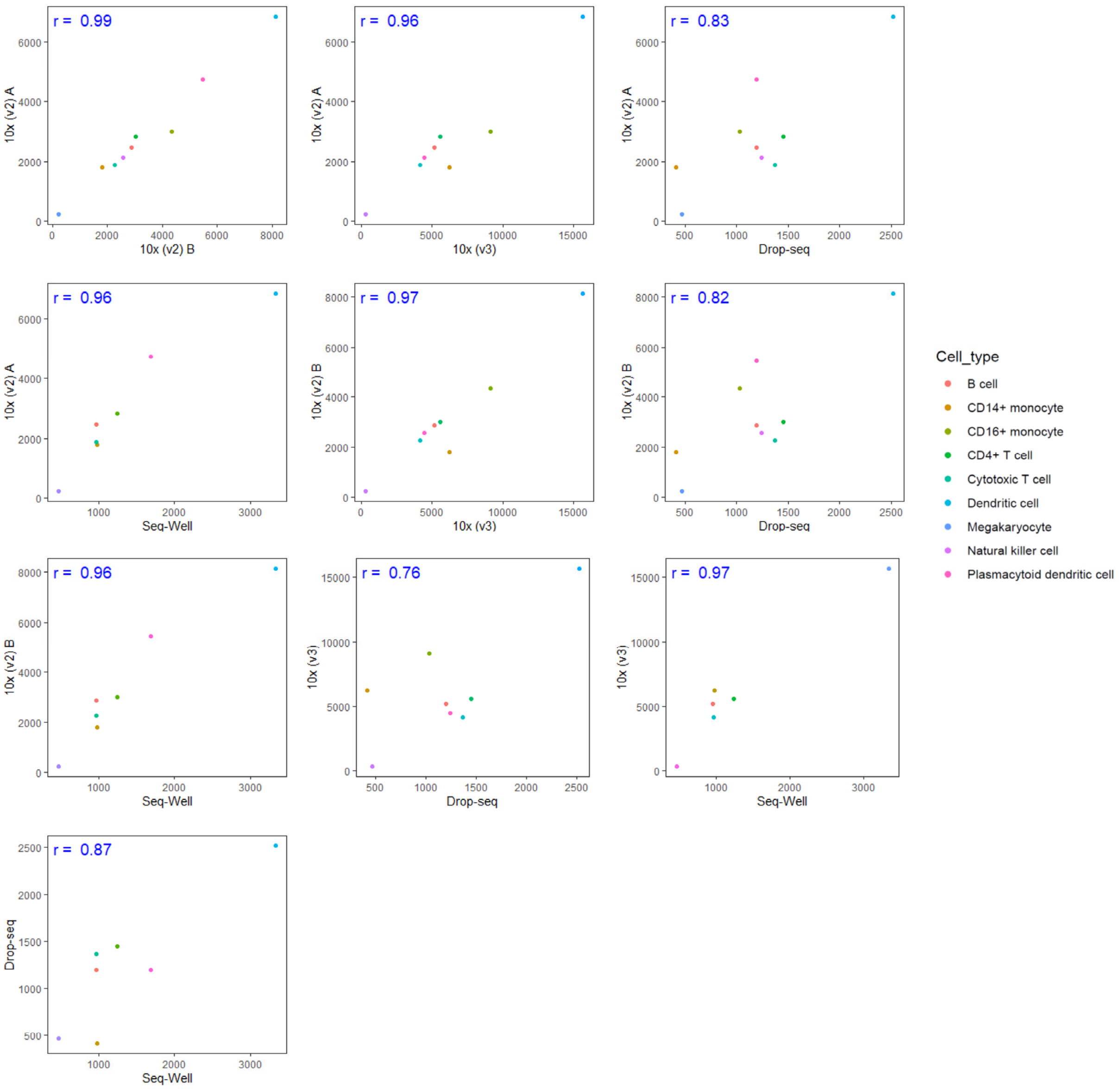

b

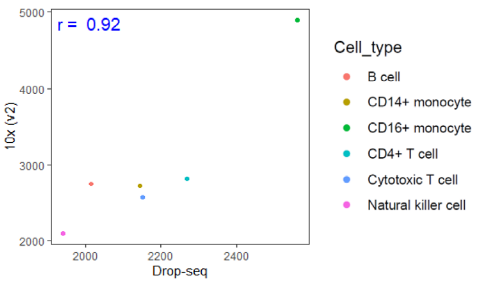

Supplementary Fig. 4 | Comparison of scRNA-seq data obtained through different protocols. **a**, **b**, Scatter plot of cell type transcriptome size means for scRNA-seq data from different platforms for samples pbmc1 (**a**) and pbmc2 (**b**). Source data are provided as a Source Data file.

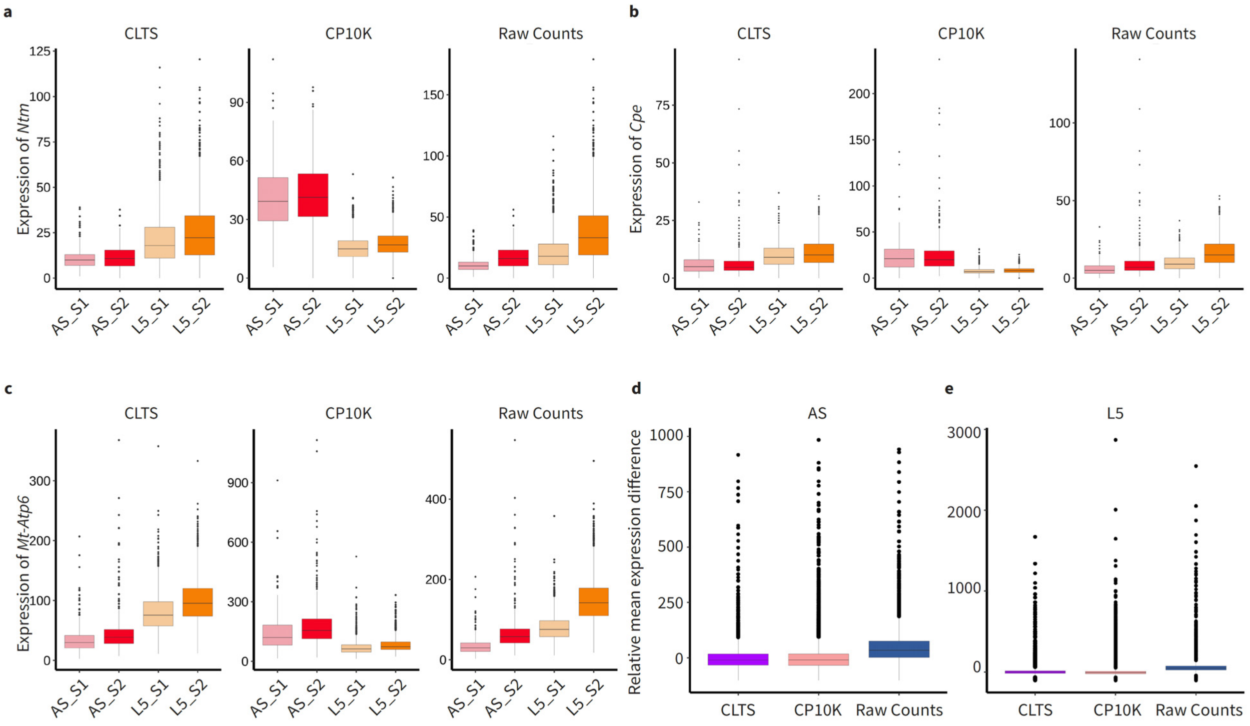

Supplementary Fig. 5 | Expression changes under CLTS, CP10K, and no normalization of scRNA-seq data. a-c, Expressions of genes, *Ntm* (**a**), *Cpe* (**b**), and *Mt-Atp6* (**c**), in L5 and AS of mouse brain Sample_I and Sample_II under CLTS-normalized, CP10K-normalized, and raw count scRNA-seq data. **d, e,** Relative differences of all genes between mouse brain Sample_I and Sample_II for AS (**d**) or L5 (**e**) under CLTS-normalized, CP10K-normalized, and raw count scRNA-seq data, respectively. In the box plots presented in this figure, the values are depicted as the median, represented by the middle line, and the 25th and 75th percentiles, represented by the box. In **a-c**, AS: S1 (n = 206), S2(n = 461); L5: S1(n = 1665), S2(n = 1857). In **d**, n = 15386. In **e**, n = 19867. Source data are provided as a Source Data file. Source data are provided as a Source Data file.

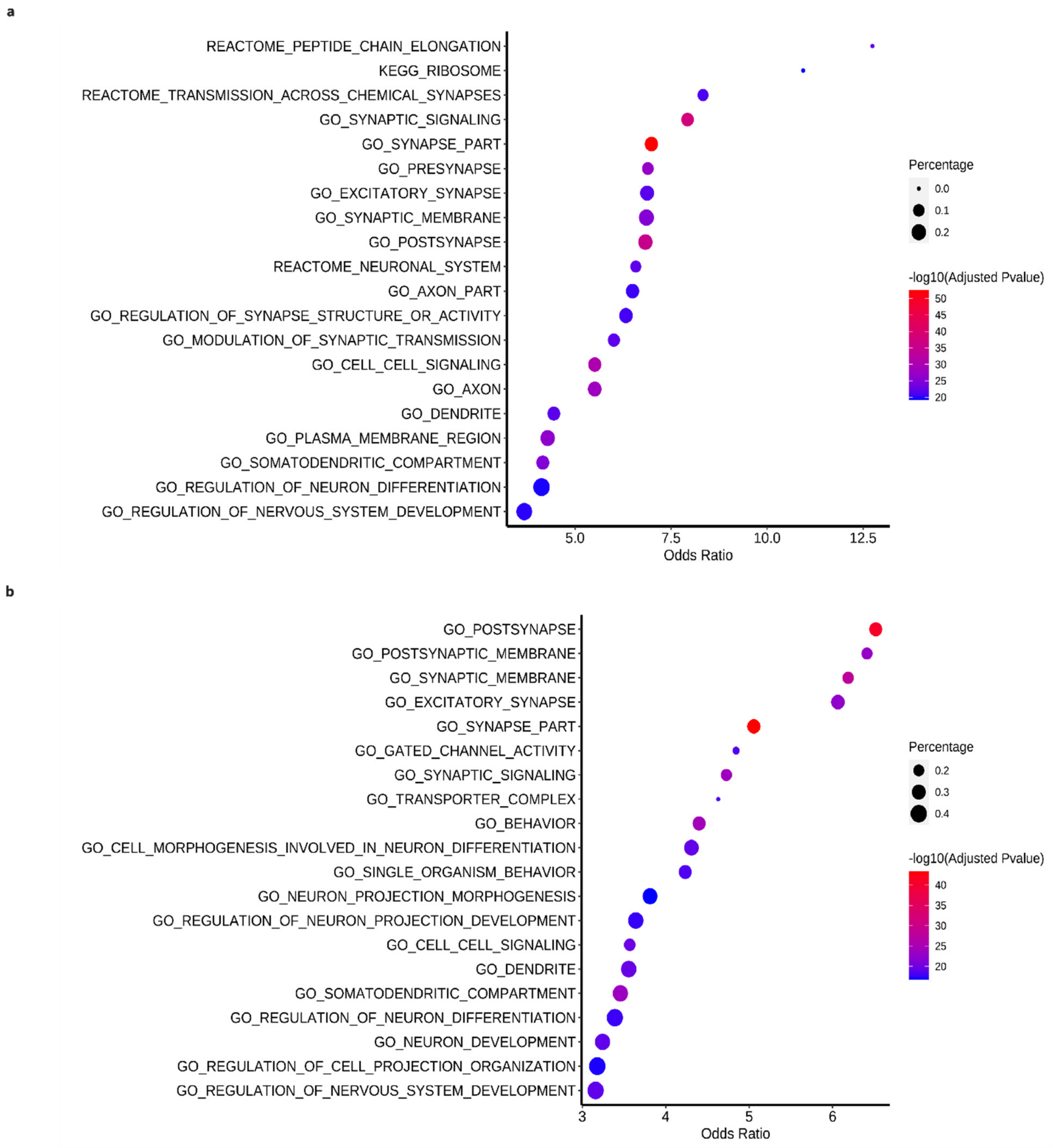

Supplementary Fig. 6 | Comparative Functional Analysis of Significantly Differentially Expressed Genes under CLTS-Normalized. a, b, These figures present the top enriched Gene Ontology (GO) terms and pathways for significant DEGs that were identified under CLTS normalization. **a,** Highlights the genes predicted to be significantly up-regulated in the L5 of mouse brain, when compared to the AS. **b,** Depicts

the genes predicted to be significantly up-regulated in the L2/3 of human brain, in comparison to the AS. Source data are provided as a Source Data file.

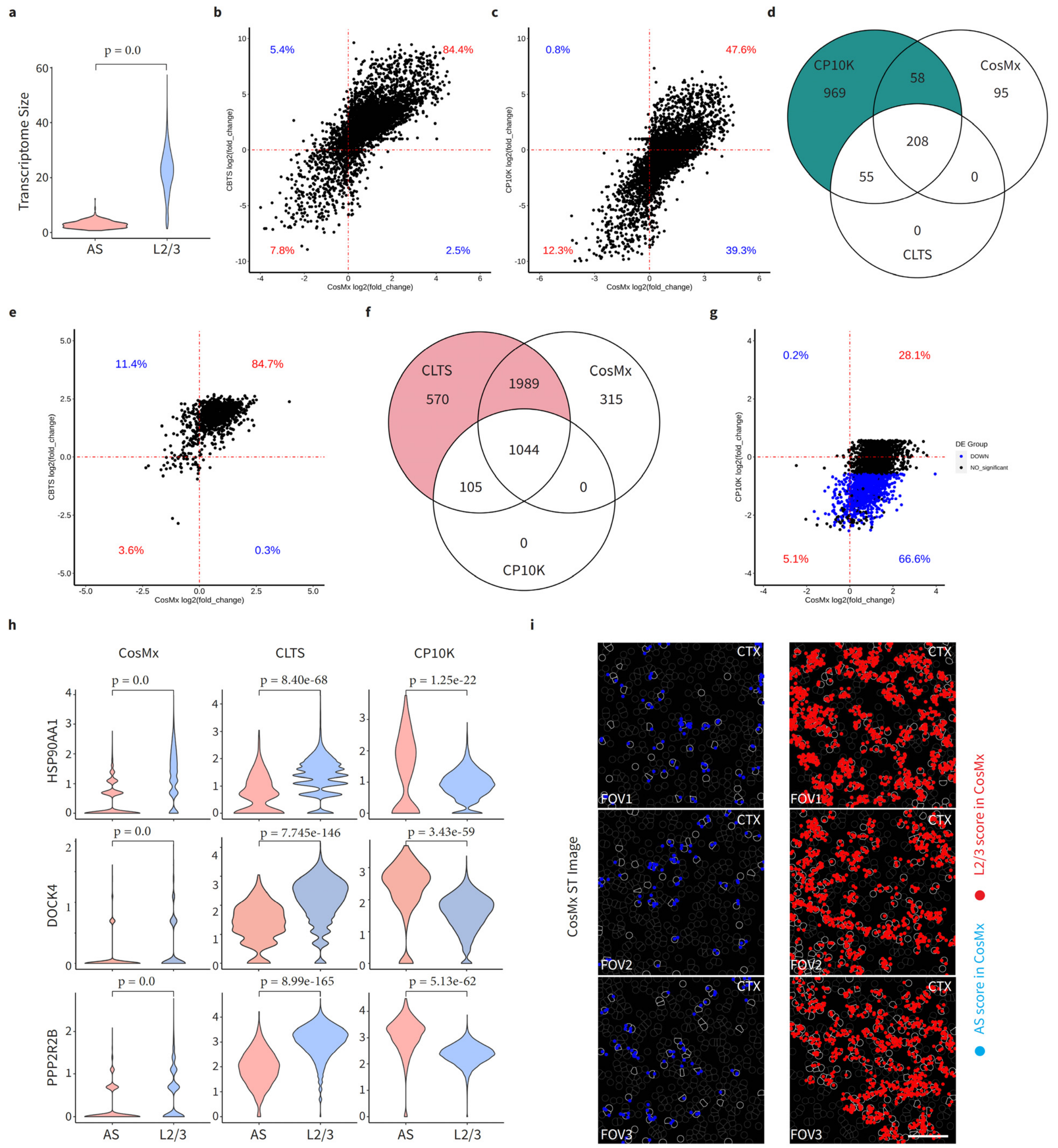

Supplementary Fig. 7 | CLTS corrects CP10K-misidentified differentially expressed genes from scRNA-seq data of human brains using CosMx data as ground truth. a, Transcriptome sizes of human brain AS and L2/3. **b, c,** Scatter plots comparing the fold-changes of genes in CosMx data and scRNA-seq

data under CLTS-normalization (**b**) and CP10K-normalization (**c**). **d,** Overlap of significantly down-regulated genes in L2/3 (vs. AS) under CosMx data, CLTS-normalized and CP10K-normalized scRNA-seq data. **e,** Scatter plots comparing the fold-changes of genes in CosMx data and scRNA-seq data under CLTS-normalization, where these genes were identified as significantly down-regulated in L2/3 (vs. AS) under CP10K-normalized but not CLTS-normalized scRNA-seq data. **f,** Overlap of significantly up-regulated genes in L2/3 (vs. AS) under CosMx data, CLTS-normalized and CP10K-normalized scRNA-seq data. **g,** Scatter plots comparing the fold-changes of genes in CosMx data and scRNA-seq data under CP10K-normalization, where these genes were identified as significantly up-regulated in L2/3 (vs. AS) under CLTS-normalized but not CP10K-normalized scRNA-seq data. **h,** Expression of *HSP90AA1*, *DOCK4*, and *PPP2R2B* in AS and L2/3 under CosMx data, CLTS-normalized, and CP10K-normalized scRNA-seq data, respectively. **i,** The actual gene signals in CosMx images for genes in **h**. In the violin plots showcased in this figure, the P-values were computed using a two-tailed t-test. In **a**, AS (n = 387), L2/3 (n = 11109). In CosMx of **h**: L2/3 (n = 14449), AS (n = 22162). In CP10K, CLTS of **h**: L2/3 (n = 11109), AS (n = 387). Source data are provided as a Source Data file.

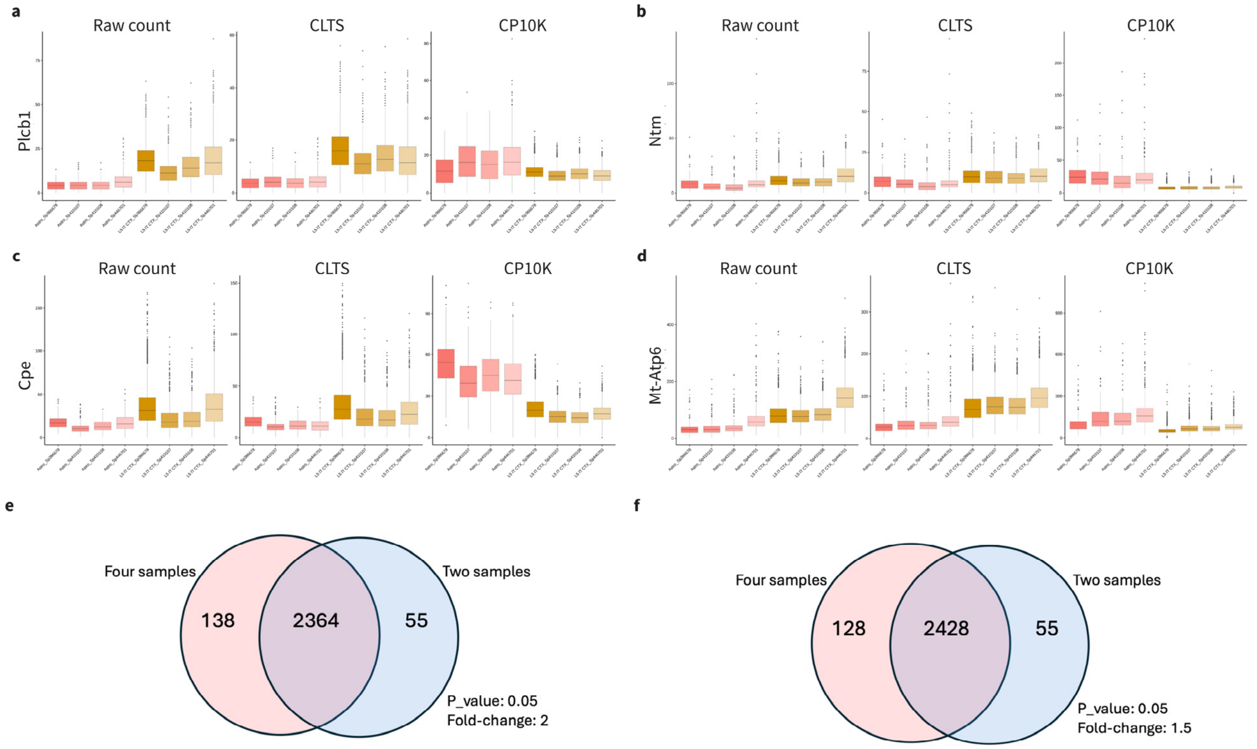

Supplementary Fig. 8 | Compare the CLTS, CP10K normalizations to scRNA-seq data with four mouse samples. **a-d,** Expressions of genes, Plcb1(a), Ntm (b), Cpe (c), and Mt-Atp6 (d), in L5 and AS of four mouse brain Samples under CLTS-normalized, CP10K-normalized, and raw count scRNA-seq data. **e-f**, overlap of up-regulated genes (L5 vs. Astro) obtained from CLTS normalized scRNA-seq data with four and two samples, respectively. In the box plots presented in this figure, the values are depicted as the median, represented by the middle line, and the 25th and 75th percentiles, represented by the box. In **a-d**, Astro: Sp410107 (n = 618), Sp410108 (n = 429), Sp446701 (n = 1383), Sp366678 (n = 408); L5 IT CTX: Sp410107 (n = 4995), Sp410108 (n = 3945), Sp446701 (n = 5571), Sp366678 (n = 12015). Source data are provided as a Source Data file.

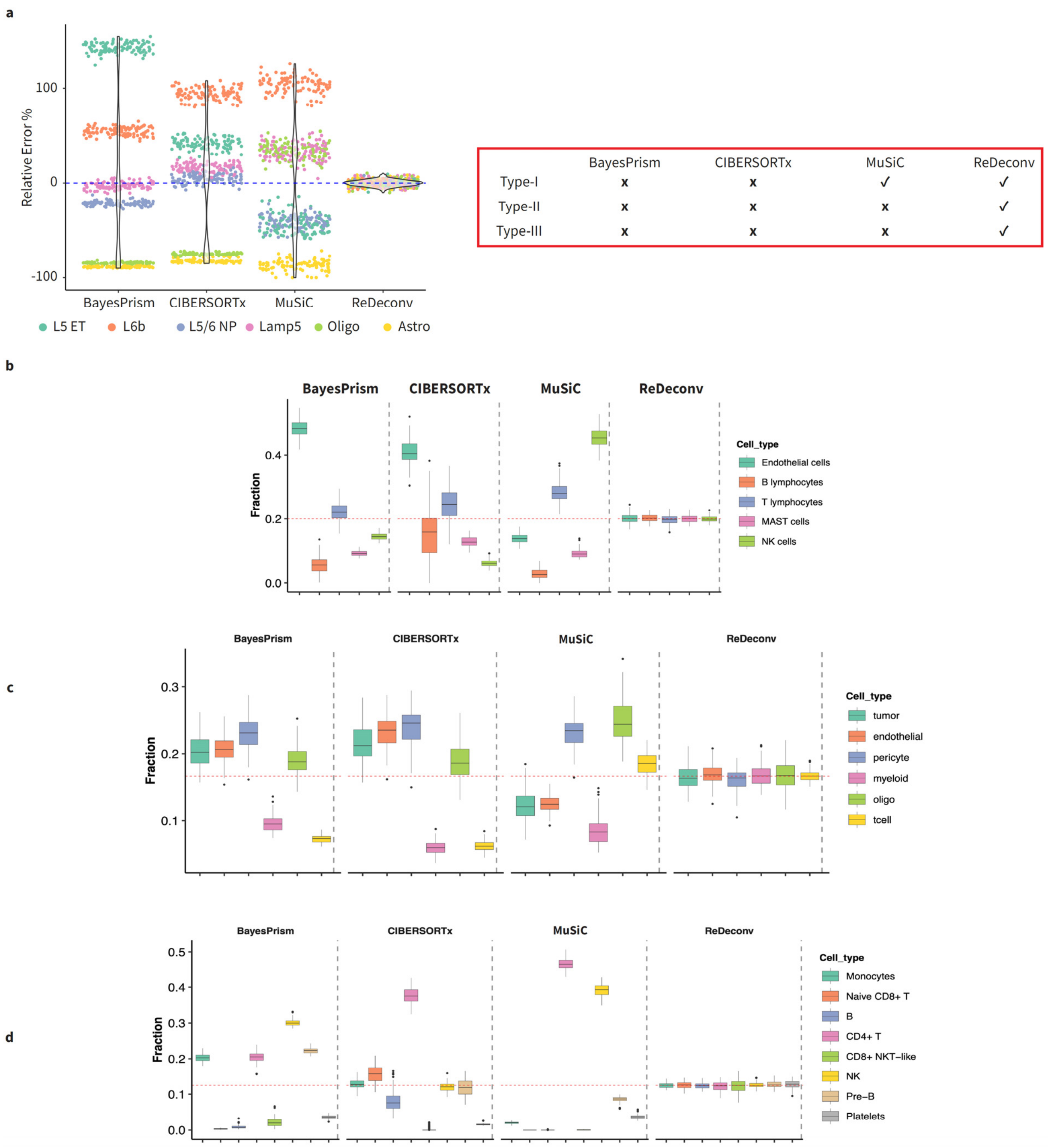

Supplementary Fig. 9 | Overall performance of ReDeconv vs. popular bulk deconvolution methods in additional synthetic datasets. a, Relative errors of prediction from different methods on SYN Data A. **b-d,** Deconvolution outcomes of BayesPrism, CIBERSORTx, MuSiC, and ReDeconv, as per the recommended input data formats outlined in their respective manuals, using synthetic bulk RNA-seq data

with equal fractions for all cell types: SYN Data B (**b**), C (**c**) and D (**d**). In these evaluations, BayesPrism and CIBERSORTx exhibited Type-I, Type-II, and Type-III issues, while MuSiC presented Type-II and Type-III issues. Notably, ReDeconv didn't display any of these three types of issues. In the box plots presented in this figure, the values are depicted as the median, represented by the middle line, and the 25th and 75th percentiles, represented by the box. In **a-d**, n=100 for each cell type. Source data are provided as a Source Data file.

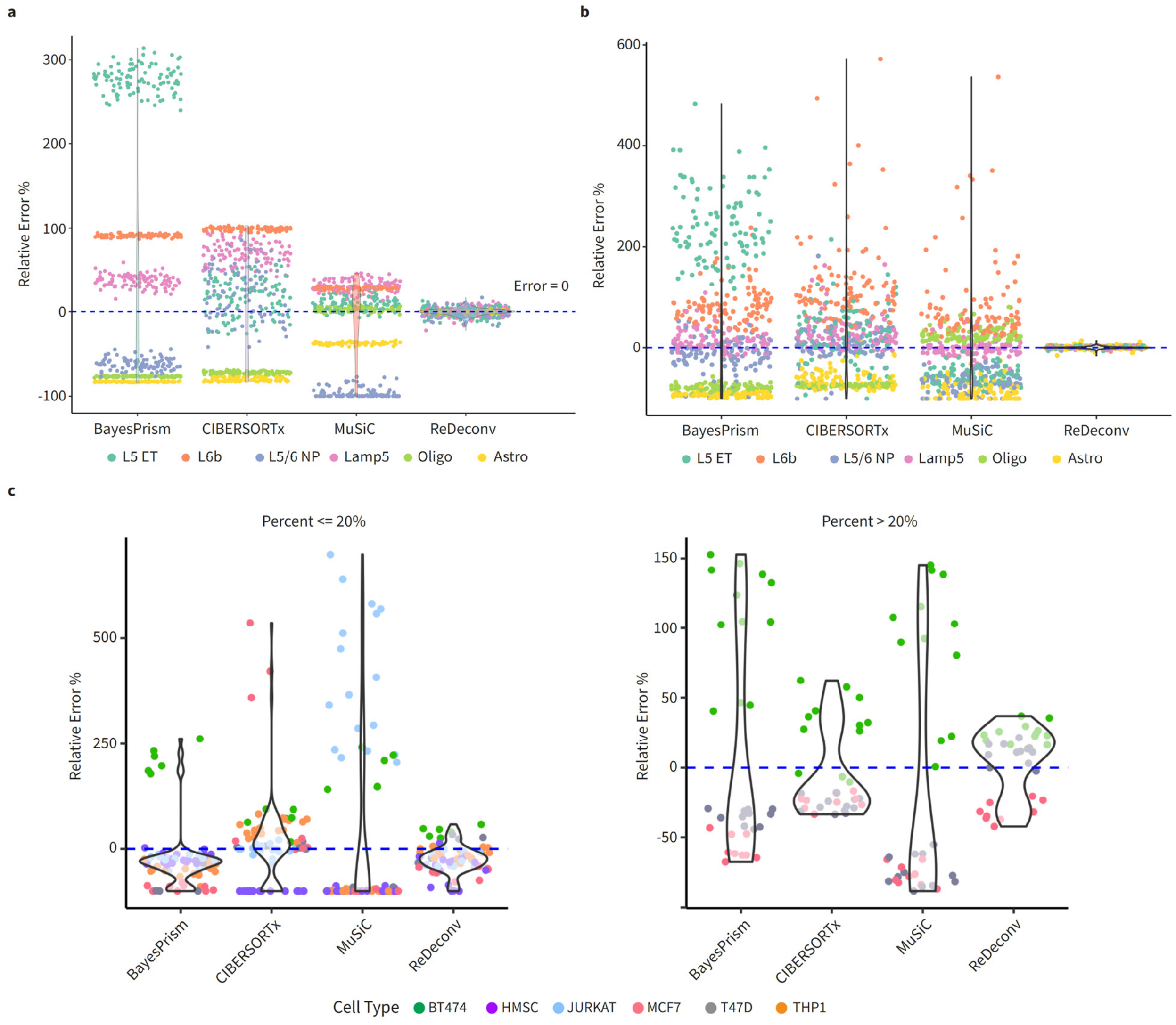

Supplementary Fig. 10 | Evaluation of relative errors in deconvolution by ReDeconv and other methods. a-b, The relative errors of predictions by different methods, according to the manuals' suggested input data format, using SYN Data E (**a**) and SYN Data F (**b**). **c**, The relative prediction errors of abundant (with fraction >20%) and rare (fraction <= 20%) cell types in real bulk RNA-seq data (GSE220606) predicted by each method. In **a-b**, n = 100 for each cell type. Source data are provided as a Source Data file.

	BayesPrism (I, III)	CIBERSORTx (I, III)	MuSiC (I, III)	ReDeconv (I)
Type-I	x	x	x	x
Type-II	✓	✓	✓	✓
Type-III	x	x	x	✓

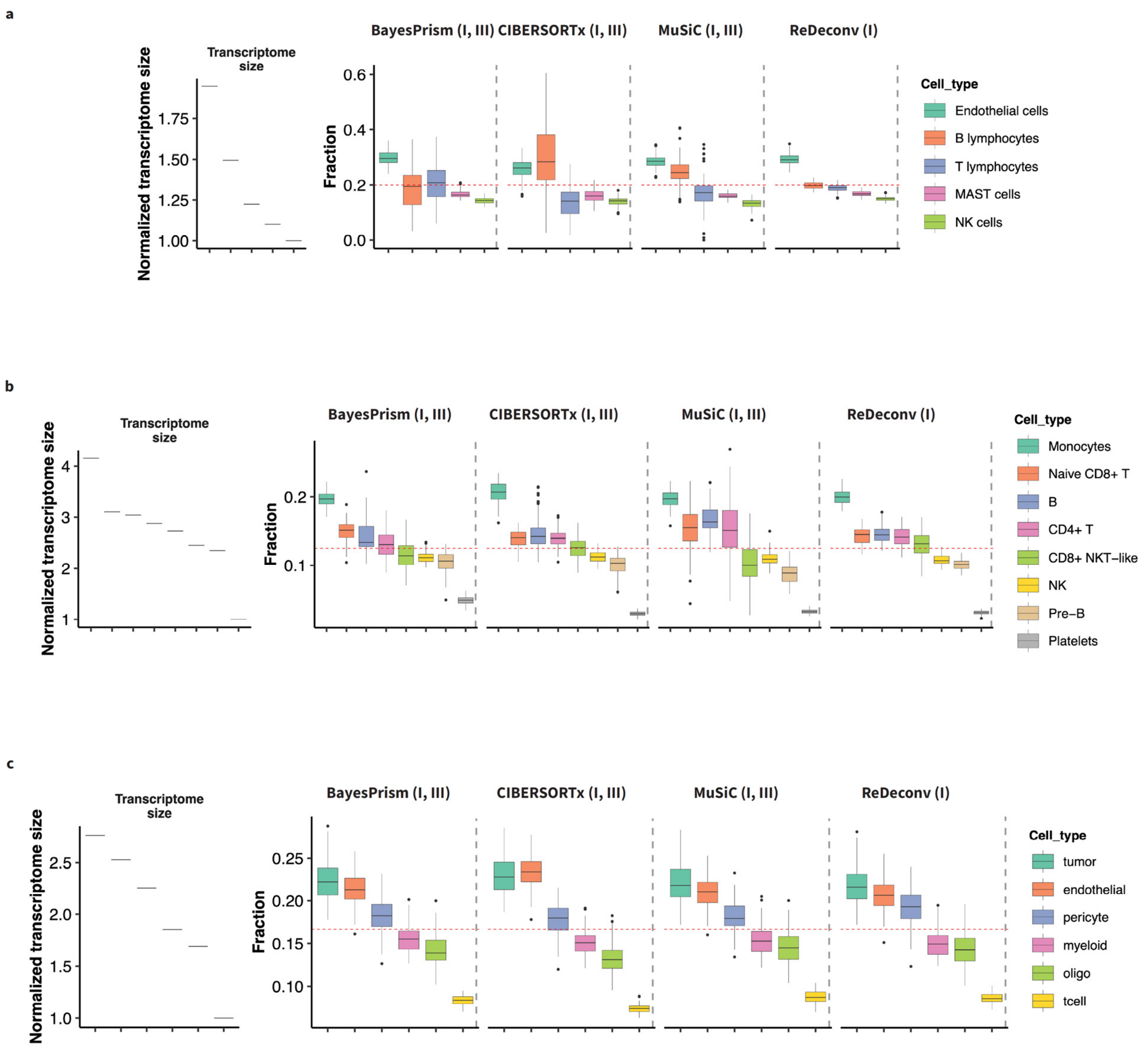

Supplementary Fig. 11 | Examining the effects of Type-I issues on bulk deconvolution. The impact of Type-I issues on BayesPrism, CIBERSORTx, MuSiC, and ReDeconv, utilizing three synthetic bulk RNA-seq data in which each cell type had equal fractions: SYN Data B (**a**), SYN Data C (**b**), and SYN Data D (**c**). In these experiments, we opted for various combinations of scRNA-seq and bulk RNA-seq data as inputs for all models, guaranteeing that each model exhibits Type-I issues, while completely eliminating

Type-II issues. BayesPrism, CIBERSORTx, and MuSiC all suffered from the Type-III issues. In the box plots presented in this figure, the values are depicted as the median, represented by the middle line, and the 25th and 75th percentiles, represented by the box. In **a-c**, n = 100 for each cell type. Source data are provided as a Source Data file.

	ReDeconv	ReDeconv (I)	ReDeconv (II)	ReDeconv (I, II)
Type-I	✓	x	✓	x
Type-II	✓	✓	x	x

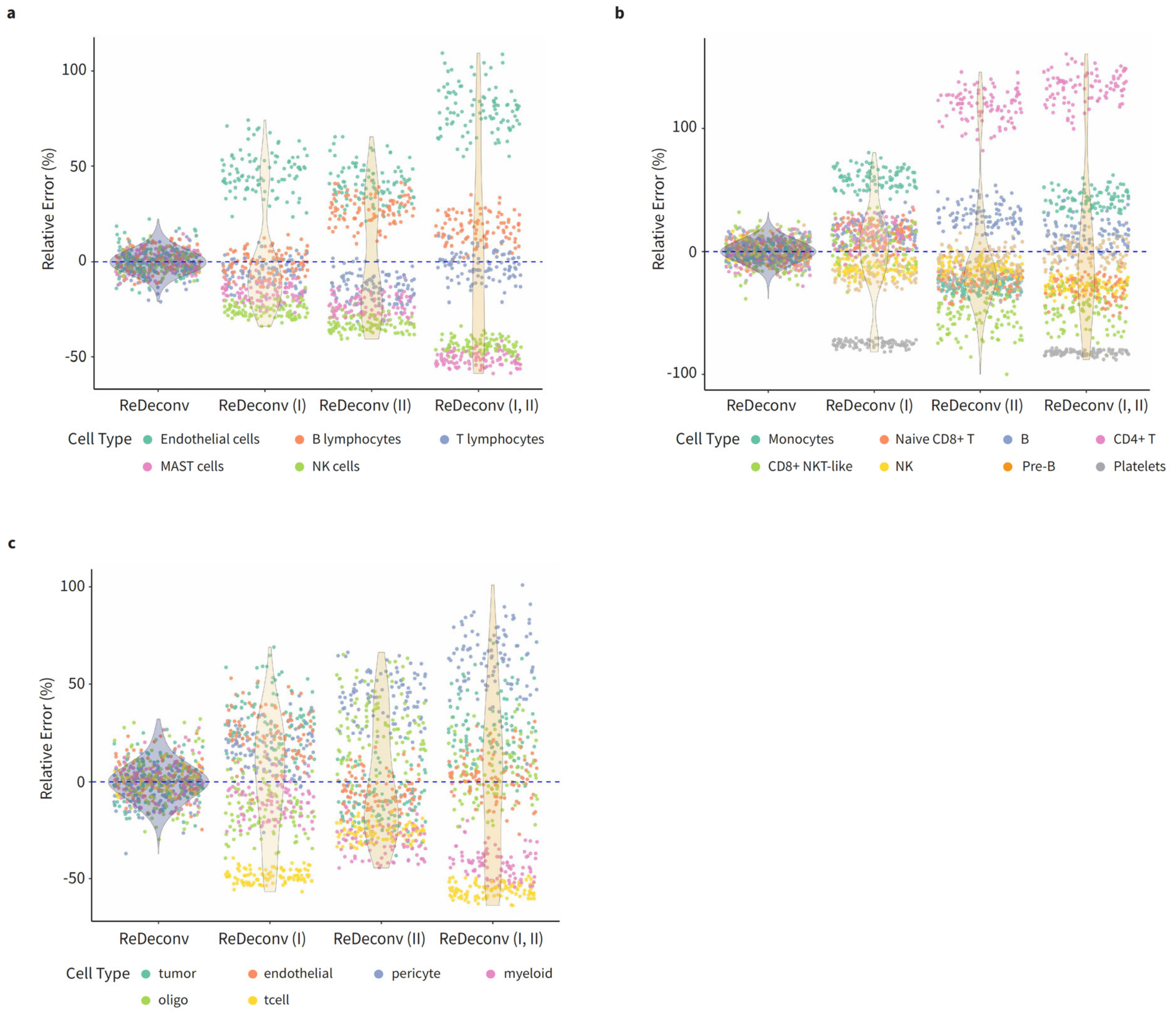

Supplementary Fig. 12 | Examining the efforts of Type-I and/or Type-II issues on bulk deconvolution using ReDeconv. Relative prediction errors of ReDeconv, ReDeconv(I) (with Type-I issues only), ReDeconv (II) (with Type II issues only), ReDeconv (I, II) (with both Type-I and Type-II issues) using three synthetic data sets: SYN Data B (**a**), SYN Data C (**b**), and SYN Data D (**c**). In **a-c**, n = 100 for each cell type. Source data are provided as a Source Data file.

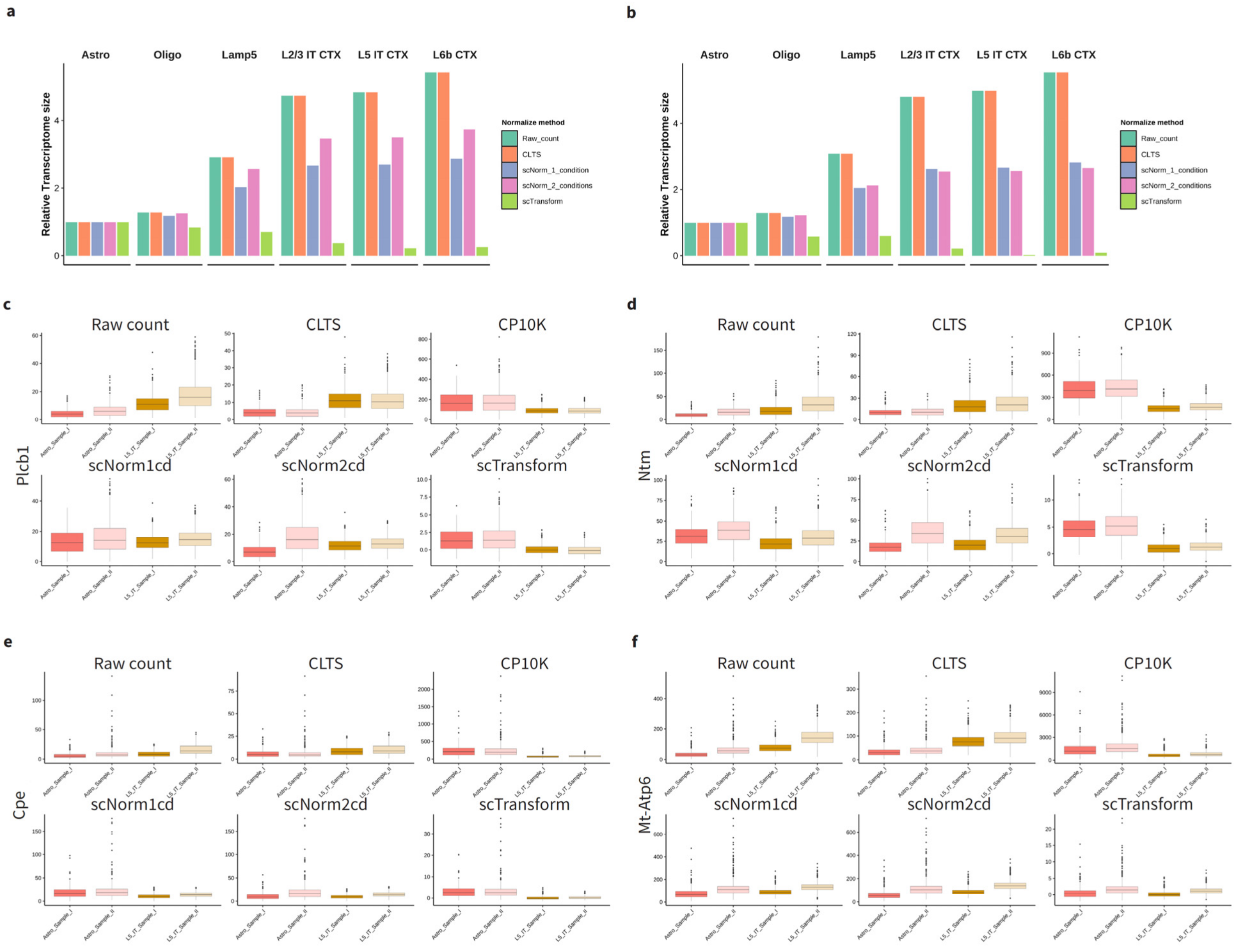

Supplementary Fig. 13 | How scaling effect impacts the scRNA-seq data normalization. a-b, Cell type transcriptome size means relative to Astro cells in mouse Sample-I (a) and Sample-II (b) under raw-count, CLTS, scNorm (assuming two samples are in identical and varying conditions), and scTransform normalized scRNA-seq data. **c-f,** Expressions of genes, Plcb1(c), Ntm (d), Cpe (e), and Mt-Atp6 (f), in L5 and AS of mouse brain Sample_I and Sample_II under raw-count, CLTS, CP10K, scNorm (assuming two samples are in identical and varying conditions), and scTransform normalized scRNA-seq data. In the box plots presented in this figure, the values are depicted as the median, represented by the middle line, and the 25th and 75th percentiles, represented by the box. In c-f, Sample_I: Astro (n = 206), L5_IT (n = 500), Sample_II: Astro (n = 461), L5_IT (n = 500). Source data are provided as a Source Data file.

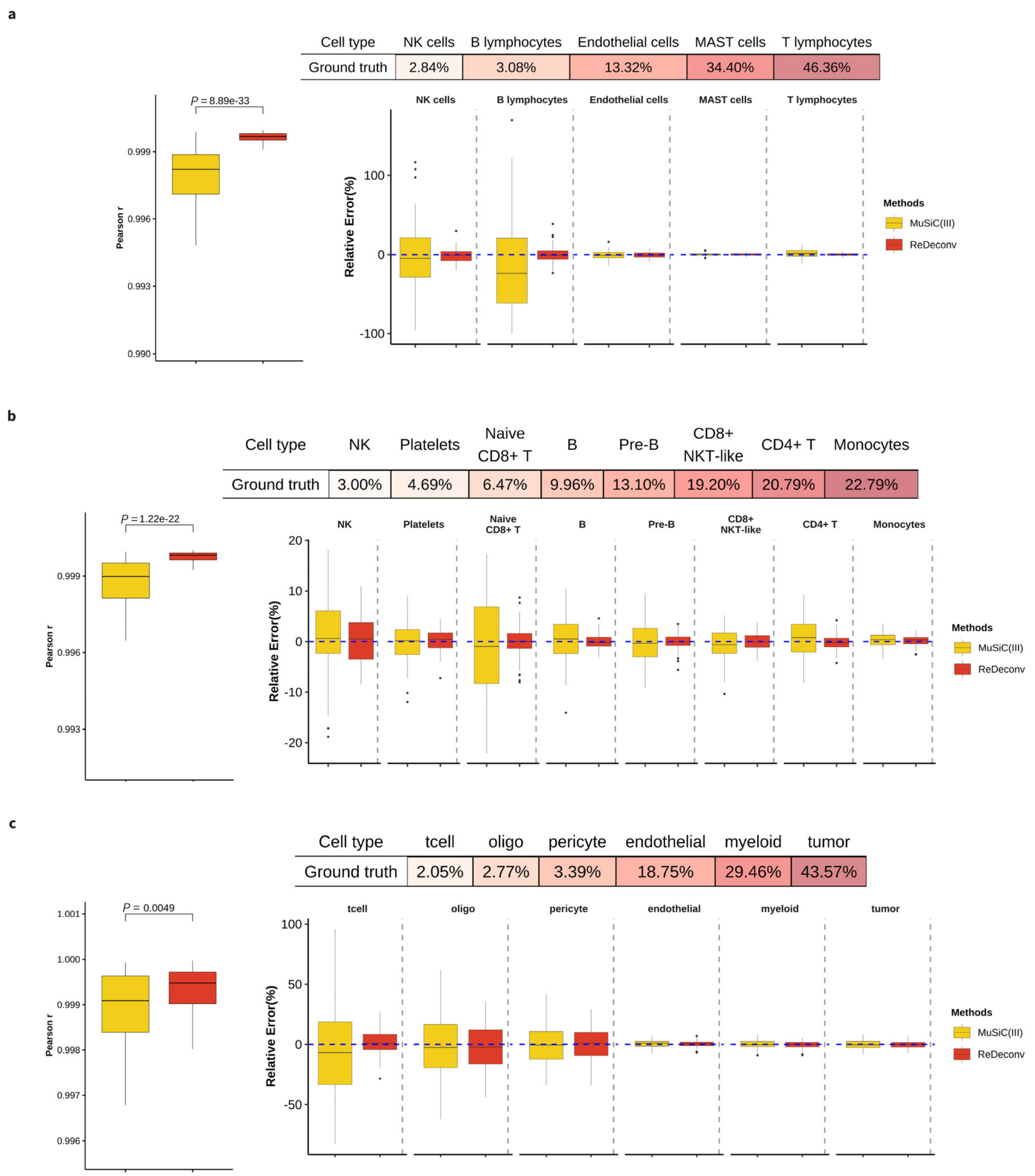

Supplementary Fig. 14 | Examining the efforts of Type-III issues on bulk deconvolution using synthetic data. Performance of MuSiC (III) (modified MuSiC with Type-III issues only) and ReDeconv

in cell type deconvolution across three synthetic bulk RNA-seq datasets: **a,** SYN Data G; **b,** SYN Data H; and **c,** SYN Data I. The distinguishing factor in these tests between the modified MuSiC and ReDeconv is that MuSiC does not account for Type-III issues. In the box plots presented in this figure, the values are depicted as the median, represented by the middle line, and the 25th and 75th percentiles, represented by the box. For the comparison of the Pearson Correlation coefficient 'r' between two populations in Figures 6a and 6b, the P-values were determined using a two-sided Wilcoxon rank-sum test. In **a-c**, n = 100 for each cell type. Source data are provided as a Source Data file.

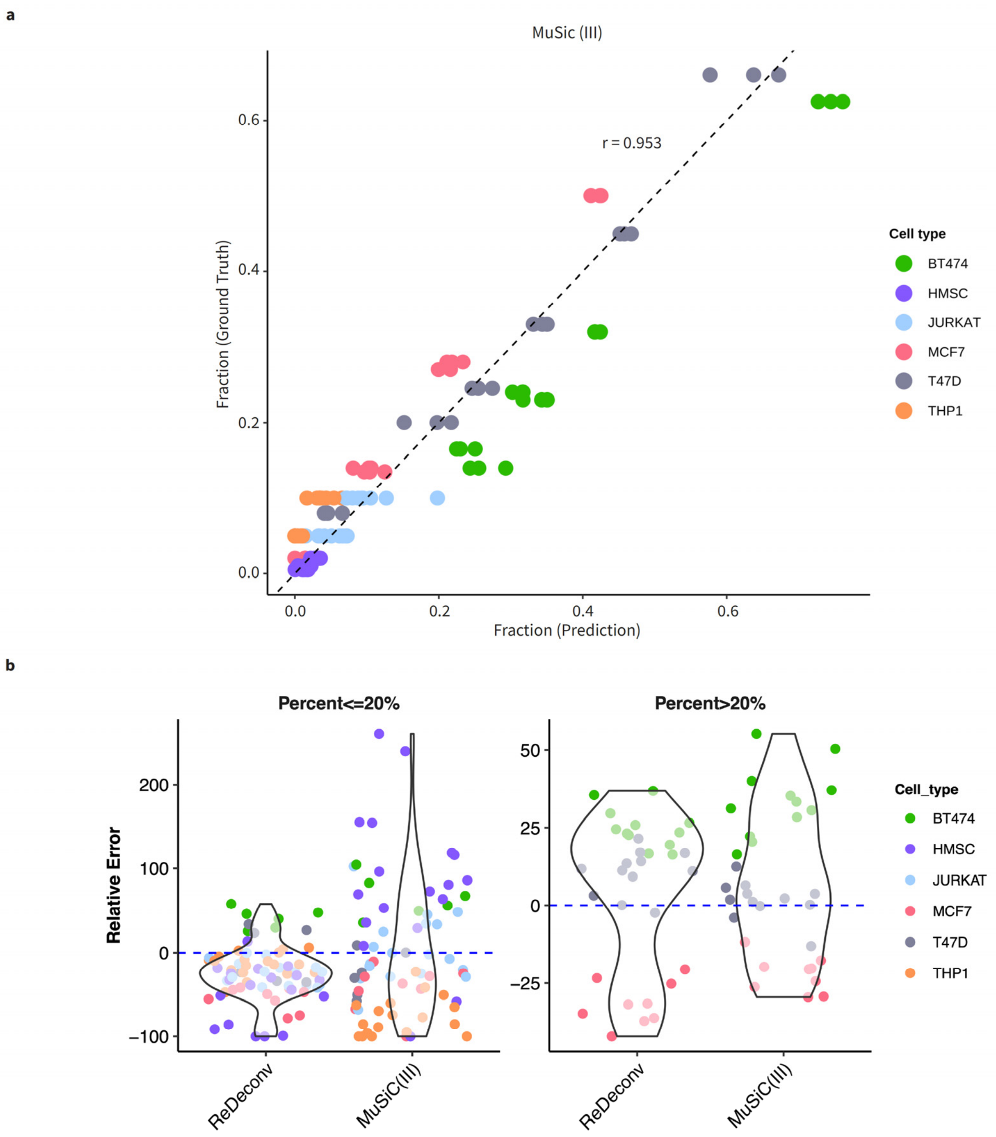

Supplementary Fig. 15 | Evaluating the effects of Type-III Issues on bulk deconvolution using real data. Performance of ReDeconv vs. MuSiC (III) (modified MuSiC with Type-III issues but no Type-I and Type-II issues) using the real data of 6 cell lines. **a,** Predicted fractions by MuSiC (III) against those from the ground truth. **b,** Relative error in predictions from ReDeconv and MuSiC (III). The left plot represented cell types with ground truth percentages not exceeding 20%, while the right plot focused on cell types with ground truth percentages surpassing 20%. Source data are provided as a Source Data file.

	BayesPrism (I, III)	CIBERSORTx (I, III)	ReDeconv (I)	MuSiC (III)	ReDeconv
Type-I	x	x	x	✓	✓
Type-II	✓	✓	✓	✓	✓
Type-III	x	x	✓	x	✓

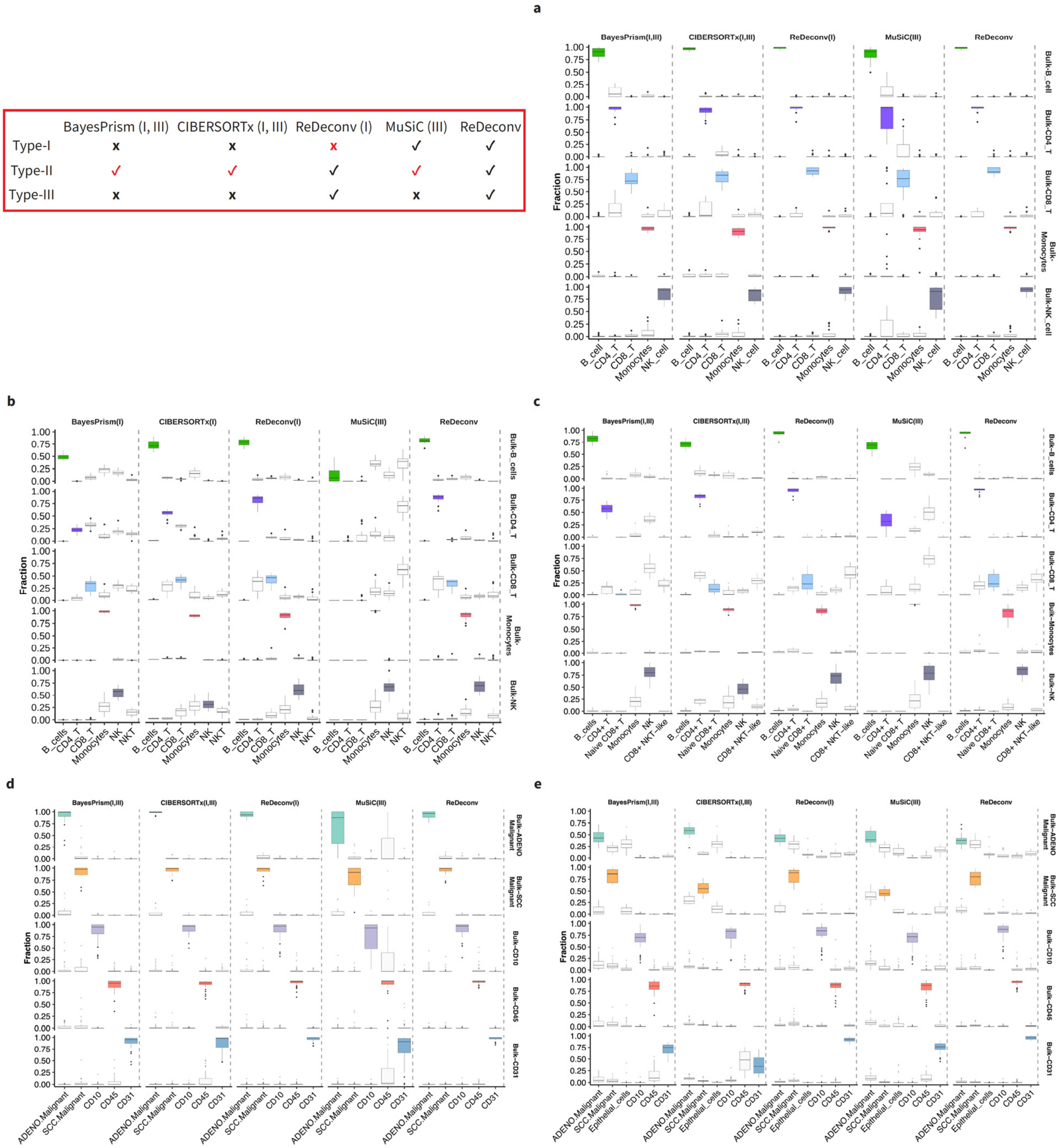

Supplementary Fig. 16 | Examining the effects of Type-III issues on bulk deconvolution using another real bulk RNA-seq dataset with different scRNA-seq references. Performance of ReDeconv against modified versions of BayesPrism, CIBERSORTx, ReDeconv, and MuSiC, using various reference and bulk sort RNA-seq datasets as inputs. In all tests, both BayesPrism (I, III) and CIBERSORTx (I, III) presented Type-I and Type-III issues, while MuSiC (III) showed Type-III issues. ReDeconv was free from these

issues. In these bulk sorted RNA-seq data sets, Type-I issues did not significantly affect the predictions of the dominant cell types. **a-c,** The mixture samples were from the bulk sort RNA-seq data of COVID-19 viremia patients (GSE216529). The reference was the same bulk sort RNA-seq data (**a**), scRNA-seq data of NSCLC PBMC sample (CIBERSORTx) (**b**), and the scRNA-seq data of health PBMC samples (CIBERSORTx) (**c**). **d, e,** the Bulk sorted RNA-seq data of NSCLC patients (GSE111907) was used as mixture samples. The reference was the same bulk sort (**d**) and the scRNA-seq data of NSCLC patients (GSE207422) (**e**). In the box plots presented in this figure, the values are depicted as the median, represented by the middle line, and the 25th and 75th percentiles, represented by the box. In **a-c**, n = 17 for each cell type. In **d-e**, CD10 (n = 34), CD31 (n = 38), CD45 (n = 40), ADENO.Malignant (n = 21), SCC.Malignant (n = 13). Source data are provided as a Source Data file.

Supplementary Table 1. Variance information of fraction predictions. Variance of relative errors for predictions from MuSiC (III) and ReDeconv; P-values from two-sides F-test for relative errors of predictions from MuSiC (III) and ReDeconv.

Match Fig. 6a

	Variances		P-values (F-test, two sides)
Cell type	MuSiC (III)	ReDeconv	MuSiC (III) vs. ReDeconv
L5.6.NP	9.03E-06	3.70E-06	1.33E-05
L5.ET	1.07E-05	3.36E-06	2.04E-08
Lamp5	1.31E-05	5.66E-06	3.69E-05
Astro	9.99E-05	1.68E-05	2.22E-16
Oligo	4.54E-05	3.20E-05	0.082465149
L6b	9.14E-05	2.24E-05	1.98E-11

Match Fig. 6b

	Variances		P-values (F-test, two sides)
Group	MuSiC (III)	ReDeconv	MuSiC (III) vs. ReDeconv
Percent<=5%	1.07E+02	1.61E+01	2.22E-16
Percent: 5%~10%	2.01E+01	4.16E+00	2.22E-16
Percent: 10%~20%	4.38E+00	1.53E+00	2.13E-08
Percent>20%	8.70E-01	4.00E-01	4.62E-08

Match supp. Fig. 14a

	Variances		P-values (F-test, two sides)
Cell type	MuSiC (III)	ReDeconv	MuSiC (III) vs. ReDeconv
NK cells	1.21E-04	5.527E-06	2.22E-16
B lymphocytes	3.06E-04	1.077E-05	2.22E-16
Endothelial cells	4.17E-05	2.044E-05	0.000460568
MAST cells	3.67E-05	2.427E-05	0.040926209
T lymphocytes	5.46E-04	5.459E-05	2.22E-16

Match supp. Fig. 14b

	Variances		P-values (F-test, two sides)
Cell type	MuSiC (III)	ReDeconv	MuSiC (III) vs. ReDeconv
NK	4.75E-06	1.89E-06	7.00E-06
Platelets	3.54E-06	9.578E-07	3.50E-10
Naive CD8+ T	3.67E-05	3.52E-06	2.22E-16
B	1.79E-05	1.787E-06	2.22E-16
Pre-B	2.52E-05	3.944E-06	2.22E-16
CD8+ NKT-like	3.41E-05	1.047E-05	1.15E-08
CD4+ T	6.90E-05	9.417E-06	2.22E-16
Monocytes	1.04E-05	4.708E-06	0.000106607

Match supp. Fig. 14c

	Variances		P-values (F-test, two sides)
Cell type	MuSiC (III)	ReDeconv	MuSiC (III) vs. ReDeconv
tcell	6.22E-05	4.491E-06	2.22E-16
oligo	5.83E-05	2.533E-05	4.51E-05
pericyte	3.13E-05	2.111E-05	0.050693995
endothelial	2.73E-05	2.344E-05	0.453378727
myeloid	1.06E-04	8.38E-05	0.249202642
tumor	2.29E-04	1.43E-04	0.020611711

Supplementary Table 2. Information on different technologies for scRNA-seq and bulk RNA-seq data. Expression raw-count, normalized value, and other information for different scRNA-seq and bulk RNA-seq methods. Suppose Rg is the actual number of RNAs expressed in a sample or cell for gene g, and Lg is the length of gene g. The **measured value** of a gene in a platform or method is the expression value of the gene observed in the platform or method.

<table>
<tr><th>method</th><th>Population</th><th>UMI</th><th>Fragmentated size</th><th>Single/Paired end</th><th>Measure value (raw count)</th><th>Normalized value (TPM, RPKM, FPKM)</th><th>Amplification</th><th>note</th></tr>
<tr><td rowspan="2">Bulk*</td><td>Total RNA w/o rRNA</td><td>No</td><td>Yes, 100-500</td><td>Single</td><td>cRgLg</td><td>cRg</td><td>Yes</td><td>Limited effects</td></tr>
<tr><td>mRNA</td><td>Yes</td><td>Yes, 100-500</td><td>Paired</td><td>cRg</td><td>cRg/Lg</td><td>Yes</td><td>Limited effects</td></tr>
<tr><td>Spatial Transcriptomics Visium v1</td><td rowspan="2">mRNA</td><td rowspan="2">Yes</td><td>Yes, 300-600</td><td rowspan="2">Paired</td><td rowspan="2">cRg</td><td rowspan="2">cRg/Lg</td><td rowspan="2">Yes</td><td rowspan="2">Limited effects</td></tr>
<tr><td>Spatial Transcriptomics Visium v2</td><td>Yes, 200-300</td></tr>
<tr><td>SMART-Seq v1/2</td><td>mRNA</td><td>No</td><td>No, full length</td><td></td><td>cRg</td><td>cRg/Lg</td><td>Yes</td><td>Limited effects</td></tr>
<tr><td>SMART-Seq v3/4</td><td>mRNA</td><td>Yes</td><td>No, full length</td><td></td><td>cRg</td><td>cRg/Lg</td><td>Yes</td><td>But no effects</td></tr>
<tr><td>CEL-Seq</td><td>mRNA</td><td>Yes</td><td>Yes, 100-500</td><td></td><td>cRg</td><td>cRg/Lg</td><td>Yes</td><td>But no effects</td></tr>
<tr><td>Drop-Seq</td><td>mRNA</td><td>Yes</td><td>Yes, 100-500</td><td></td><td>cRg</td><td>cRg/Lg</td><td>Yes</td><td>But no effects</td></tr>
<tr><td>10X</td><td>mRNA</td><td>Yes</td><td>Yes, 100-500</td><td></td><td>cRg</td><td>cRg/Lg</td><td>Yes</td><td>But no effects</td></tr>
</table>

*---	Illumina	HiseSq
		NextSeq
		NovaSeq
		MiSeq
*---	Affymetrix	Human Gene
		Huaman Genome
		Huaman Exon
		Clariom

Supplementary Table 3: Type of issues four methods may have in different combinations of inputs. **Note**: The highlight represents the recommended settings from methods' manuals.

	scRNA-seq: raw-count/CLTS<br>bulk RNA-seq: raw count	scRNA-seq: CPM<br>bulk RNA-seq: raw count	scRNA-seq: raw-count/CLTS<br>bulk RNA-seq: TPM	scRNA-seq: CPM<br>bulk RNA-seq: TPM
BayesPrism	Type-I, II, III	Type-I, II, III	Type-I, III	Type-I, III
CIBERSORTx	Type-I, II, III	Type-I, II, III	Type-I, III	Type-I, III
MuSiC	Type-II, III	Type-I, II, III	Type-III	Type-I, III
ReDeconv	Type-II	Type-I, II		Type-I
