# Supplementary material for: Transcriptome size matters for single-cell RNA-seq normalization and bulk deconvolution
Source: Nat Commun. 2025 Feb 1;16:1246. doi: 10.1038/s41467-025-56623-1 (PMC11787294; doi:10.1038/s41467-025-56623-1)
Supplement: Supplementary file 2 — Description of Additional Supplementary Files [file 41467_2025_56623_MOESM2_ESM.pdf]

Description of Additional Supplementary Files:

Supplementary Data 1: Information of expression data for mixture samples and references. Information of data source, sample number, data type, and normalization status for all scRNA-seq and bulk RNA-seq data for evaluation.

Supplementary Data 2: Ground truth for synthetic bulk RNA-seq data. Information of fractions for all cell types in synthetic bulk RNA-seq data for evaluation.
