# Supplementary material for: Transcriptome size matters for single-cell RNA-seq normalization and bulk deconvolution
Source: Nat Commun. 2025 Feb 1;16:1246. doi: 10.1038/s41467-025-56623-1 (PMC11787294; doi:10.1038/s41467-025-56623-1)
Supplement: Supplementary file 4 — Reporting Summary [file 41467_2025_56623_MOESM4_ESM.pdf]

nature portfolio

Corresponding author(s): Jiyang Yu

Last updated by author(s): 1/5/2025

Reporting Summary

Nature Portfolio wishes to improve the reproducibility of the work that we publish. This form provides structure for consistency and transparency in reporting. For further information on Nature Portfolio policies, see our Editorial Policies and the Editorial Policy Checklist.

Statistics

For all statistical analyses, confirm that the following items are present in the figure legend, table legend, main text, or Methods section.

n/a	Confirmed	
☐	☒	The exact sample size (*n*) for each experimental group/condition, given as a discrete number and unit of measurement
☒	☐	A statement on whether measurements were taken from distinct samples or whether the same sample was measured repeatedly
☐	☒	The statistical test(s) used AND whether they are one- or two-sided<br>*Only common tests should be described solely by name; describe more complex techniques in the Methods section.*
☒	☐	A description of all covariates tested
☒	☐	A description of any assumptions or corrections, such as tests of normality and adjustment for multiple comparisons
☐	☒	A full description of the statistical parameters including central tendency (e.g. means) or other basic estimates (e.g. regression coefficient) AND variation (e.g. standard deviation) or associated estimates of uncertainty (e.g. confidence intervals)
☐	☒	For null hypothesis testing, the test statistic (e.g. *F*, *t*, *r*) with confidence intervals, effect sizes, degrees of freedom and *P* value noted<br>*Give P values as exact values whenever suitable.*
☒	☐	For Bayesian analysis, information on the choice of priors and Markov chain Monte Carlo settings
☒	☐	For hierarchical and complex designs, identification of the appropriate level for tests and full reporting of outcomes
☐	☒	Estimates of effect sizes (e.g. Cohen's *d*, Pearson's *r*), indicating how they were calculated

Our web collection on statistics for biologists contains articles on many of the points above.

Software and code

Policy information about availability of computer code

Data collection	We gathered publicly accessible single cell RNA-seq and bulk RNA-seq data, from which we generated synthetic bulk RNA-seq data. Details of all the data utilized for evaluation are available in Supplementary Table S3. The procedure for generating synthetic bulk RNA-seq data is outlined in the Supplementary Information section of the manuscript.
Data analysis	We conducted a comparative analysis of our ReDeconv model with three other cell type deconvolution models, namely CIBERSORTx, BayesPrism, and MuSiC, using both synthetic and real bulk RNA-seq data. All these programs are publicly accessible and can be located in their respective referenced publications.<br>CIBERSORTx[https://cibersortx.stanford.edu/index.php]: a web portal for cell type deconvolution and expression profile purification of bulk RNA-seq data.<br>MuSiC[https://github.com/xuranw/MuSiC]: a deconvolution method that utilizes scRNA-seq data of multiple subjects to estimate cell type percentage of bulk RNA-seq data.<br>BayesPrism [https://github.com/Danko-Lab/BayesPrism]: deconvolution method that uses scRAN-seq data as reference to find percentages of cell types in bulkRNA-seq data.<br>ReDeconv [https://github.com/jyyulab/redeconv]: a new method for cel type deconvolution that addresses critical issues overlooked by current existing methods.

For manuscripts utilizing custom algorithms or software that are central to the research but not yet described in published literature, software must be made available to editors and reviewers. We strongly encourage code deposition in a community repository (e.g. GitHub). See the Nature Portfolio guidelines for submitting code & software for further information.

Data

Policy information about availability of data

All manuscripts must include a data availability statement. This statement should provide the following information, where applicable:

- Accession codes, unique identifiers, or web links for publicly available datasets
- A description of any restrictions on data availability
- For clinical datasets or third party data, please ensure that the statement adheres to our policy

The authors declare that all scRNA-seq, synthetic and real bulk RNA-seq data used for evaluations are available at https://redeconv.stjude.org.

Research involving human participants, their data, or biological material

Policy information about studies with human participants or human data. See also policy information about sex, gender (identity/presentation), and sexual orientation and race, ethnicity and racism.

Reporting on sex and gender	N/A
Reporting on race, ethnicity, or other socially relevant groupings	N/A
Population characteristics	N/A
Recruitment	N/A
Ethics oversight	N/A

Note that full information on the approval of the study protocol must also be provided in the manuscript.

Field-specific reporting

Please select the one below that is the best fit for your research. If you are not sure, read the appropriate sections before making your selection.

☒ Life sciences ☐ Behavioural & social sciences ☐ Ecological, evolutionary & environmental sciences

For a reference copy of the document with all sections, see nature.com/documents/nr-reporting-summary-flat.pdf

Life sciences study design

All studies must disclose on these points even when the disclosure is negative.

Sample size	Nine synthetic bulk RNA-seq data sets with 100 samples in each data set. Three real bulk RNA-seq data with 185, 85, and 6 samples, respectively.
Data exclusions	Given the data size constraints of CIBERSORTx, we facilitated unbiased comparisons by randomly selecting cell subsets from single cell RNA-seq data when the data sizes were overly large. These selected data subsets were then applied across all methods for evaluation purposes.
Replication	We verifed the performance of ReDeconv on different real and synthetic datasets.
Randomization	N/A
Blinding	N/A

Reporting for specific materials, systems and methods

We require information from authors about some types of materials, experimental systems and methods used in many studies. Here, indicate whether each material, system or method listed is relevant to your study. If you are not sure if a list item applies to your research, read the appropriate section before selecting a response.

Materials & experimental systems

n/a	Involved in the study
☒	☐ Antibodies
☒	☐ Eukaryotic cell lines
☒	☐ Palaeontology and archaeology
☒	☐ Animals and other organisms
☒	☐ Clinical data
☒	☐ Dual use research of concern
☒	☐ Plants

Methods

n/a	Involved in the study
☒	☐ ChIP-seq
☒	☐ Flow cytometry
☒	☐ MRI-based neuroimaging

Plants

Seed stocks	N/A
Novel plant genotypes	N/A
Authentication	N/A
